# The Role of Environmental Transmission in Recurrent Avian Influenza Epidemics

**DOI:** 10.1371/journal.pcbi.1000346

**Published:** 2009-04-10

**Authors:** Romulus Breban, John M. Drake, David E. Stallknecht, Pejman Rohani

**Affiliations:** 1 Odum School of Ecology, University of Georgia, Athens, Georgia, United States of America; 2 Southeastern Cooperative Wildlife Disease Study, University of Georgia, Athens, Georgia, United States of America; 3 Center for Tropical and Emerging Global Diseases, University of Georgia, Athens, Georgia, United States of America; 4 Fogarty International Center, National Institutes of Health, Bethesda, Maryland, United States of America; Imperial College London, United Kingdom

## Abstract

Avian influenza virus (AIV) persists in North American wild waterfowl, exhibiting major outbreaks every 2–4 years. Attempts to explain the patterns of periodicity and persistence using simple direct transmission models are unsuccessful. Motivated by empirical evidence, we examine the contribution of an overlooked AIV transmission mode: environmental transmission. It is known that infectious birds shed large concentrations of virions in the environment, where virions may persist for a long time. We thus propose that, in addition to direct fecal/oral transmission, birds may become infected by ingesting virions that have long persisted in the environment. We design a new host–pathogen model that combines within-season transmission dynamics, between-season migration and reproduction, and environmental variation. Analysis of the model yields three major results. First, environmental transmission provides a persistence mechanism within small communities where epidemics cannot be sustained by direct transmission only (i.e., communities smaller than the *critical community size*). Second, environmental transmission offers a parsimonious explanation of the 2–4 year periodicity of avian influenza epidemics. Third, very low levels of environmental transmission (i.e., few cases per year) are sufficient for avian influenza to persist in populations where it would otherwise vanish.

## Author Summary

Avian influenza viruses (AIVs) in wild waterfowl constitute the historic source of human influenza viruses, having a rich pool of genetic and antigenic diversity that often leads to cross-species transmission. Although the emergence of H5N1 avian influenza virus onto the international scene has captured the most attention, we do not as yet understand the mechanisms that underpin AIV persistence and dynamics in the wild. We developed a novel host–pathogen model intended to describe the epidemiology of low pathogenic AIV in temperate environments. Our model takes into account seasonality in migration and breeding together with multiple modes of transmission. AIVs have been detected in unconcentrated lake water, soil swabs, and mud samples. Laboratory experiments show that AIVs persist and remain infectious in water for extended periods. However, so far, the possibility of environmental transmission of AIV has been largely overlooked. Our work shows that environmental transmission provides a parsimonious explanation for the patterns of persistence and outbreaks of AIV documented in the literature. In addition to their scientific importance, our conclusions impact the design of control policies for avian influenza by emphasizing the dramatic and long-term role that environmental persistence of pathogens may play at the epidemic level.

## Introduction

Many important infectious diseases persist on a knife-edge: rapid rates of transmission coupled with brief infectious periods generate boom-and-bust epidemics that court extinction. Such violent epidemic behavior has been observed in measles [1]–[4], plague [5], cholera [6], meningitis [7],[8], and pertussis [9], among others. Several distinct mechanisms have been proposed to explain the long-term dynamics and persistence of these pathogens. For example, measles persistence is primarily determined by the rate at which the susceptible pool is replenished, leading to Bartlett's concept of *critical community size*, the minimum population size above which an infectious disease remains endemic [4]. In contrast, plague is enzootic in rodents and their fleas and thus its persistence in human populations is explained by intermittent reintroduction from the animal reservoir [10]. King et. al [6] argue that rapid loss of immunity to cholera may replenish the human susceptible pool so quickly that large amplitude cholera outbreaks can be observed semiannually. Finally, rich strain polymorphism allows echoviruses –responsible for aseptic meningitis– to circumvent host immunity and thus reinvade the population [7],[8]. These examples illustrate the need for understanding alternate persistence/re-invasion mechanisms of infectious diseases for effective management and control.

In this paper, we investigate the persistence and dynamics of low pathogenic avian influenza virus (AIV) in North American bird populations. Avian influenza viruses in wild waterfowl constitute the historic source of human influenza viruses [11], with a rich pool of genetic and antigenic diversity [11],[12] that often leads to cross-species transmission. Perhaps the best-known and most topical example is the transmission of H5N1 avian influenza virus to humans [13]. Human infection with H5N1 is associated with a significant risk of mortality; to date, approximately 50% of infected individuals have died from the infection (see [13] and references therein). Developing a better understanding of the ecology of avian influenza viruses is, therefore, very timely.

AIVs infect more than 90 species of birds from 13 orders, mostly Anseriformes (ducks) and Charadriiformes (shorebirds). Long-term studies of AIV prevalence in North America [14],[15] have gathered time series of annual estimates that extend over 26 years for Anseriformes and 20 years for Charadriiformes. The data is stratified over influenza subtype: H3, H4, and H6 were the most prevalent subtypes isolated from Anseriformes. Most interestingly, the prevalence of infection with these subtypes as well as the aggregate prevalence exhibit recurrent outbreaks in duck populations at 2–4 year intervals.

It is well established that birds infected with avian influenza are infectious for approximately a week (range 6–10 days), during which they continuously shed vast concentrations of viral particles in their feces [11],[16],[17]. These virions are then ingested by susceptible birds, completing the fecal/oral transmission route [18],[19]. However, attempts to recover the patterns of periodicity and persistence in avian influenza epidemics in waterfowl from simple modeling principles using only this essentially direct transmission mechanism are unsuccessful (see, for example, Text S1 and Discussion). We propose that the missing ingredient in direct transmission models is the additional indirect contribution made to transmission by the ingestion of infectious virions that persist in the environment. It has been demonstrated, for example, that the avian influenza strain H2N4 (A/Blue-winged teal/TX/421717/01) can persist for extended periods in the environment, with an estimated one log decay time of 490 days in water at temperature 4°C, pH 7.2, salinity 0 ppt [16],[20]. Additionally, these persistent virions are known to be infectious [16],[20],[21], arguing for a potentially significant epidemiological contribution by environmental transmission.

Here we examine whether environmental transmission provides a more parsimomious explanation for the observed patterns of avian influenza epidemics. The phenomenon of environmental transmission is known to be significant for viral infections in humans (e.g., gastroenteritis [22]) and animals (e.g., rabbit haemorrhagic disease [23]), water-borne pathogens (e.g., cholera [6],[24] and avian cholera [25]), some bacterial infections (e.g., tetanus [26], salmonella [27] and epizootics of plague [28]), prion diseases (e.g., chronic wasting disease [29] and bovine spongiform encephalopathy [30]) and zoonoses (e.g., Nipah and Hendra viral diseases [31]). Despite these examples, the epidemiological consequences of environmental transmission remain poorly understood [32]–[38].

Here we propose a new host-pathogen model that combines within-season transmission dynamics, with a between-season component that describes seasonal migration, reproduction and environmental variation. Analysis of deterministic and stochastic versions of this model shows that environmental transmission plays a critical role for the persistence of avian influenza and its inter-annual epidemics. We conclude that environmental transmission may provide a parsimonious explanation of the observed epidemic patterns of avian influenza in wild waterfowl.

## Model

**Figure 1:**
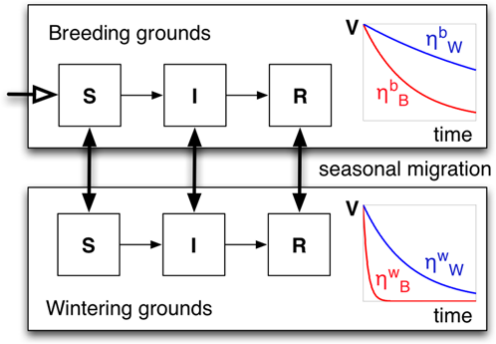
Illustration of the model. The decay curves of the virus during winter and summer are sketched in blue and red, respectively. The corresponding symbols of the viral persistence rates within each ground are also illustrated. The persistence rates of avian influenza strains in the breeding and wintering grounds are quite different because they increase strongly with the temperature of the environment. Since water temperatures where the ducks are present (i.e., breeding grounds in the summer and wintering grounds in the winter) may be similar, we chose the corresponding persistence rates to be similar, as well. The persistence rate is much reduced (i.e., the persistence time of the virus increases) in the breeding grounds during the winter as the temperature drops. Also, the persistence rate is significantly increased (i.e., the persistence time of the virus decreases) in the wintering grounds during the summer as the temperature increases.

Our model is designed to represent a typical population (∼5,000–10,000 individuals) of ducks (Anseriformes) that migrates twice a year between a northern breeding ground and a southern wintering ground. As shown in Figure 1, the model assumes two geographically distinct sites linked by rapid migration (thick black arrows). The duration of the breeding and the wintering seasons are assumed to be the same. At the beginning of each breeding season, new susceptible chicks are added to the flock (Figure 1, open thick arrow); i.e., we assume pulsed reproduction.

Given the uncertain and possibly complex patterns of cross-immunity in wild ducks, we focus on the dynamics of a single subtype. Hence, we assume that after recovery from infection, ducks acquire life-long immunity. Thus, within each season, the epidemiological dynamics are of the familiar 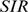 type with two transmission routes: direct and environmental. To derive the environmental transmission functional form, we denote the probability that a duck escapes infection when exposed to 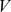 virions by 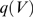; note that 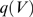 must decrease with 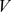 and 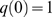. Next, we consider a bird that is exposed to 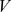 virions in two steps: first 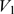 virions and then 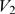 virions 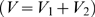. Therefore, 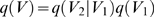 where 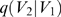 is the conditional probability that the duck will escape infection when exposed to 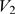 virions after escaping infection when exposed to 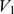 virions. It is assumed that there is no immunological consequence of unsuccessful exposure; that is, the probability of escaping infection is independent of past AIV challenges that did not result in infection (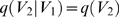). Thus, we obtain the exponential Cauchy equation [39] 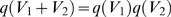. Since 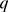 is a decreasing probability function defined on all non-negative real numbers, the only acceptable solution is 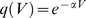 where 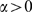 is a constant with unit of 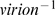. Therefore, environmental infection is modeled using a continuous Markov chain with a constant rate 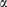. Note that the parameter 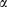 is related to the empirically determined 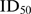 (the dose at which there is a 50% probability of infection) by the following equation 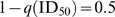, giving 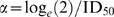. However, a bird is exposed to virus in the environment via continuous ingestion of lake water. To model this, we introduce a constant rate 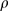 that expresses the *per capita* fraction of the 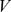 virions ingested per unit time. Thus 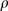, which we call *exposure rate*, is given by the *per capita* consumption rate scaled by the lake volume. The transmission rate per susceptible due to environmental contamination is given by 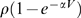.

Infected ducks shed virus in the environment where the virus persists. We assume that the viral population 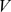 is large enough so that these two processes can be captured by the following differential equation
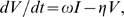
(1)where 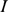 is the number of infecteds, 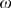 is the shedding rate and 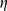 is the decay rate of the virus in the environment. If we divide the above equation by 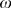 and use the variable 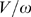 instead of 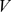 then the equation no longer contains the parameter 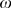. Using 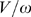 instead of 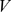 amounts to measuring the number of virions 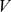 in units of 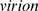 per shedding rate (i.e., 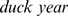) which is the unit that we adopt for the rest of the paper. The environmental transmission rate now becomes 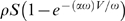, where 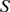 is the number of susceptibles. Thus, the dynamics of the model depends on 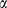 and 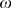 through their product 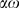, which is a re-scaled environmental infectiousness.

Model variables and parameters are presented in Tables 1 and 2, respectively. We use capital subscripts to denote the season (i.e., 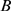 for the breeding season and 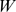 for the wintering season) and lower case superscripts for geographical location (i.e., 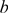 for the breeding grounds and 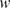 for the wintering grounds). For a deep understanding of the system, we develop two versions of the model: (i) a deterministic system, with continuous state variables, and (ii) a hybrid framework that consists of discrete population variables, and stochastic demographic and transmission transition probabilities together with deterministic virus kinetics. The transmission dynamics within the continuous model are expressed as coupled ordinary differential equations and are useful in examining the underlying deterministic clockwork of the system. Not surprisingly, however, this framework often predicts biologically unrealistic fractional numbers of infecteds (Mollison's so-called “atto-fox” phenomenon [40]). Since we are particularly interested in the processes of extinction and persistence of AIV, we further refined our study by constructing a stochastic model, where the host population variables are integer-valued.

**Table 1:** The variables of the model.

Symbol	Definition	Unit
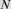	number of ducks	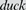
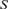	susceptible ducks	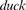
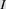	infected ducks	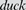
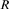	recovered ducks	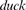
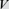	viral population	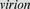
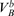	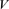 in the breeding grounds during the summer	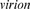
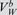	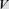 in the breeding grounds during the winter	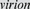
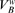	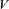 in the wintering grounds during the summer	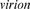
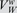	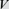 in the wintering grounds during the winter	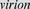

**Table 2:** The parameters of the model.

Symbol	Definition	Value/Range	Unit	Reference
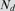	habitat carrying capacity	3000	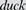	–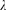	duck fecundity	2		[56],[57]
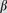	direct transmissibility	0–0.05	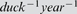	–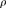	exposure rate	10^−3^	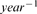	–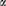	environmental infectiousness		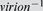	–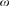	virus shedding rate	10^5^–10^6^	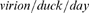	[58]
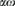	re-scaled environmental infectiousness	1–10^6^	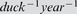	–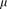	natural death rate	0.3	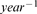	[56]
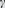	recovery rate	52	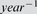	[11]
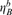	virus clearance rate in the breeding grounds during the summer	5	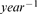	[21]
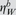	virus clearance rate in the breeding grounds during the winter	1.3	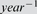	[21]
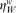	virus clearance rate in the wintering grounds during the winter	5	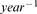	[21]
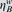	virus clearance rate in the wintering grounds during the summer	50	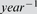	[21]

For further explanation of the parameter values see the Text S1.

### Model with continuous variables

The model proceeds as follows.

1. *The start of the Breeding Season.* We start with the initial conditions 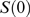, 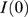, 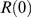, 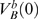 and 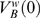 at the beginning of the breeding season. Then, we add new chicks to the flock. As with many natural reservoirs, the pathogenicity of AIV to birds is neglible, thus we assume that ducks have a fixed realized fecundity, 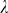, irrespective of infection history. We further assume that chick survival is density-dependent and is determined by 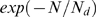, where 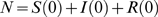 is the total number of ducks and 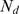 is the carrying capacity of the habitat. Therefore, the number of chicks that join the flock every breeding season is 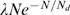; i.e., 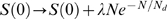.
2. *Breeding Grounds Dynamics.* We now integrate the variables 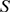, 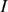, 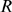, 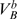 and 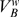 for the duration of the breeding season (i.e., half a year) according to the following set of differential equations:
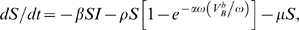
(2)

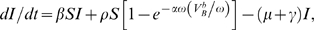
(3)

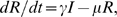
(4)

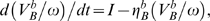
(5)

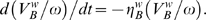
(6)The first three equations describe the well-known 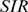 model [5], with the addition of an environmental transmission term. The last two equations describe the dynamics of the virus at the breeding and wintering grounds, respectively. They reflect the fact that during the summer at the breeding grounds, virus is shed by infected birds and decays in the environment. On the wintering grounds, however, there are no ducks during the summer, hence virion kinetics are only affected by viral degradation.
3. *Wintering Grounds Dynamics.* At the end of the breeding season, we introduce viral population variables for the wintering season 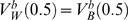 and 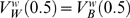 and continue the integration for another half of a year using the following set of differential equations that implicitly accounts for the migration
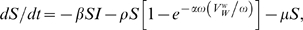
(7)

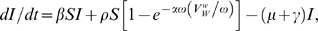
(8)

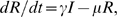
(9)

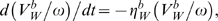
(10)

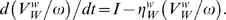
(11)At the end of the wintering season we set 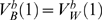 and 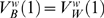 and resume with step 1. with the next breeding season in a similar fashion.

### Hybrid model

In this model, the bird population variables are discrete, evolving through a continuous-time Markov chain integrated using Gillespie's direct method [41]. The 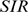 processes that take place throughout a season and their corresponding rates are summarized in Table 3. The algorithm of the model is as follows.

**Table 3:** The processes that take place within a season.

Process	Definition	Rate
Direct infection	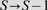 , 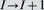	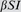
Environmental infection	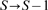 , 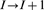	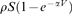
Death of susceptible	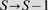	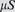
Death of infected	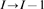	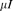
Death of recovered	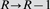	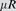
Recovery	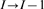 , 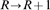	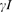

The variables and the parameters are explained in Table 1.

1. *The start of the Breeding Season.* Start with the initial conditions 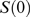, 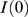, 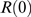, 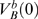 and 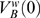 at the beginning of the breeding season. New chicks are added as before except that the number of chicks is given by a binomial stochastic variable 
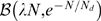
.
2. *Breeding Grounds Dynamics.* We stochastically integrate the variables 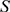, 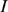 and 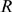 according to Gillespie's algorithm for one half of a year (i.e, one season). The variables 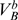 and 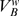 are integrated within a season using Eq. (1). For a time interval 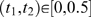 where 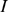 is constant,
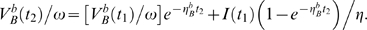
(12)For the wintering ground we get
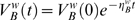
(13)where 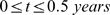, as there are no ducks at the wintering grounds.
3. *Breeding Grounds Dynamics.* At the end of the breading season, we introduce viral population variables for the wintering season 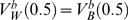 and 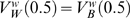. The variables 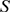, 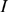 and 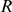 are integrated as before. 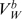 and 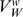 are integrated as follows. 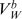 is given by
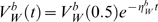
(14)where 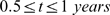, as the ducks have left the breeding grounds. 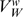 is given by
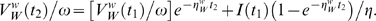
(15)for every time interval 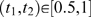 where 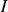 is constant. At the end of the wintering season we set 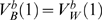 and 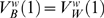 and continue with step 1. in a similar fashion.

We note that a continuous-time Markov chain where all the variables 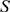, 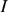 and 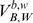 are evolved using point processes can be easily constructed by adding birth (i.e., 
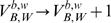
 with rate 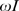 for 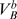 and 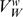 and rate 0 for 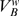 and 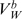) and death processes (i.e., 
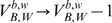
 with rate 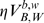) for 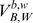 to the list presented in Table 3. First, it can be shown that if the variables of this Markov chain are approximately uncorrelated and normally distributed, then their expectations satisfy the equations of the continuous model presented in the previous section [42]; i.e., that the mean-field approximation of this Markov chain is the continuous model represented by Eqs. (2)–(11). Second, our hybrid model is a good approximation of the continuous-time Markov chain when the variables 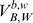 are large and the sum of their rates is much larger than the sum of all the other rates. Indeed, under these conditions, most processes are births and deaths of virions and other processes occur only sporadically. In between these processes, the stochastic dynamics of the viral load provided by the continuous-time Markov chain can be satisfactorily approximated by the deterministic equations of the hybrid model. We thus conclude that in the case where virus is abundant the continuous model represents the mean-field approximation to our hybrid model described above.

## Results

### Model without environmental transmission

As a baseline, we first explored a simplified model that includes fecal/oral transmission, migration, seasonality and pulsed reproduction, without environmental transmission. Whether stochastic or deterministic, this model is unable to reproduce the recurrent pattern of avian influenza epidemics. The continuous model shows unrealistic infected populations as low as 10^−8^ individuals (see Text S1) while the stochastic model undergoes rapid extinction when the infected population drops to zero.

### Deterministic orbits of mixed transmission model

**Figure 2:**
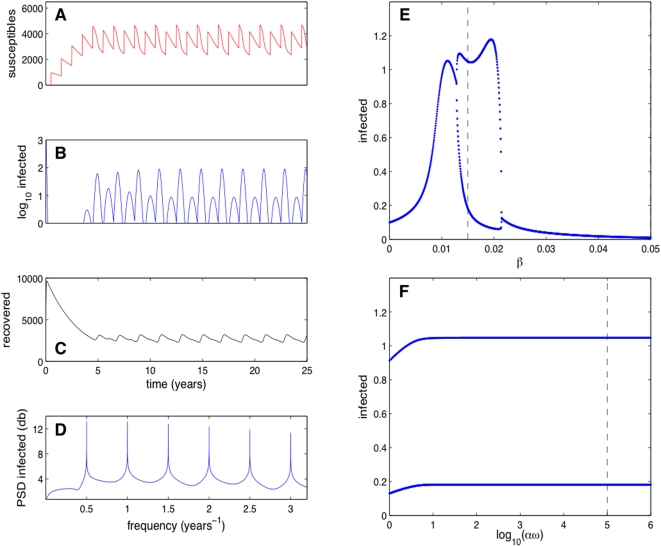
Simulation results obtained using our deterministic model. Panels A, B, and C show 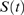, 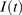 and 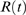 versus 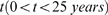; note the logarithmic scale for 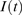. The initial conditions are 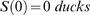, 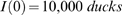, 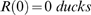, 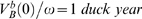 and 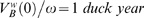. The parameters are as in Table 1 with 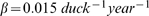 and 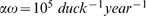. Panel D shows the Fourier power spectrum density of 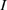 over a time interval of 25,000 years. Panels E and F show bifurcations diagrams of the model versus 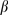 and 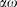, respectively. The orbits are sampled yearly, at the end of the wintering season. The dotted lines mark the positions of the orbit presented on the left within the corresponding bifurcation diagrams.

Figure 2 shows numerical results for a typical orbit of our deterministic model with both direct and environmental transmission mechanisms (for definitions of the technical terms in this section the reader is referred to [43]–[46]). The orbit rapidly settles to an attractor with a period of two years. Figure 2A–C show the number of susceptibles, infected and recovered versus time, respectively. The Fourier power spectrum density of the infected time series is presented in Figure 2D; a peak at 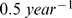 is easily noted. Figure 2E and 2F show bifurcation diagrams versus the direct transmisibility 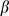 and the re-scaled environmental infectiousness 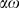, respectively. The orbits are sampled annually at the end of the wintering season when, each year, the number of infected is the lowest. Panel (e) shows a period doubling and an inverse period doubling bifurcation, while no bifurcations are present in Panel (f). The position of the orbit presented on the left is marked with dotted lines.

However, the continuous model for the parameters of avian influenza in populations of 5,000 to 10,000 individuals regularly predicts numbers of infecteds less than one. Thus, the epidemic would often go extinct as the number of infected would reach zero. This phenomenon is not captured by a continuous-state model. Furthermore, note that in Figure 2E model dynamics are predicted to be rigidly biennial, in contrast to the erratic 2–4 year outbreaks observed in the wild.

### Stochastic orbits of mixed transmission model

**Figure 3:**
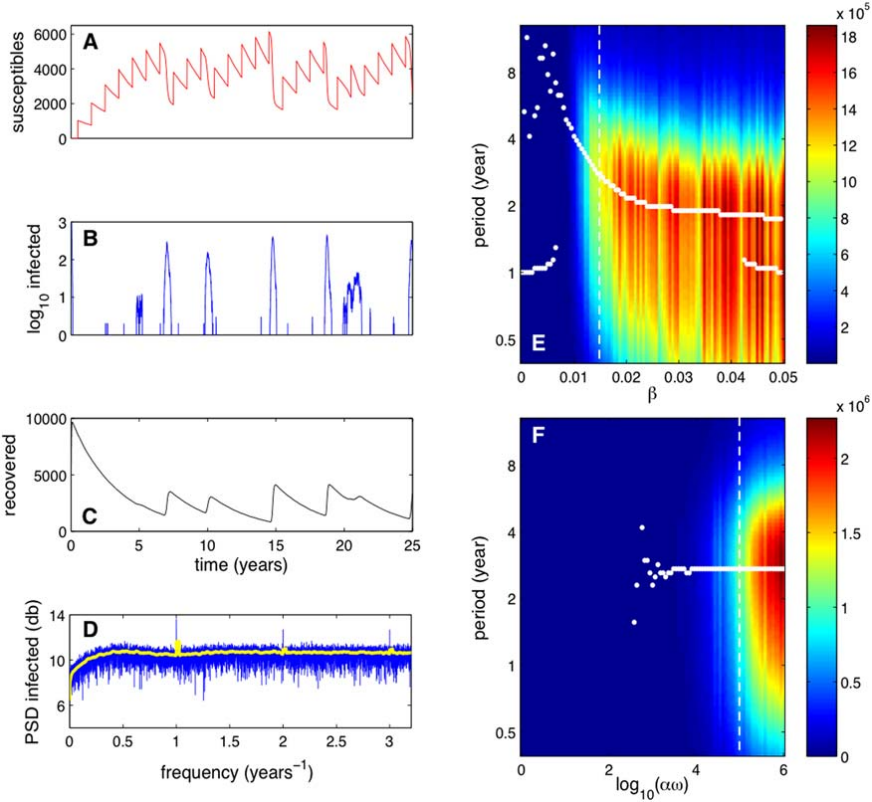
Simulation results obtained using our stochastic model. Panels A, B, and C show 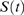, 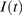 and 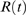 versus 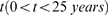; note the logarithmic scale for 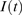. The initial conditions are 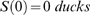, 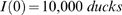, 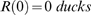, 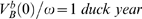 and 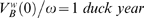. The parameters are as in Table 1 with 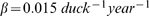 and 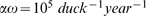. The blue line in panel D shows the Fourier power spectrum density of 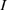 over a time interval of 3,500 years. The yellow line represents the moving average of the spectrum density. Panels E and F show the global spectral decomposition in Difference-of-Gaussians (DoG) wavelets of stochastic orbits versus 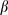 and 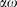, respectively. Each spectrum is an average over 100 wavelet transforms of individual stochastic realizations of the orbit over 3,300 years (this time interval gives 95% confidence to the peaks of each wavelet transform; the fluctuations are due to the stochasticity of the realizations of the model). The color map represents the power scale measured in 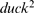. The dotted lines mark the positions of the stochastic realization presented on the left within the corresponding panels.

To understand the extinction and persistence dynamics of avian influenza we integrated the stochastic version of the model. Figure 3A–C show the number of susceptibles, infected and recovered versus time, respectively, in a simulation of our stochastic model. In this case, the infected population often goes extinct and the epidemic is then reignited by environmental transmission. In direct contrast to the predictions of the deterministic model, a major epidemic does not occur every two years as such an event is sparked probabilistically (Figure 3B). In general, the periodicity of stochastic orbits is larger than that of corresponding deterministic orbits. If an epidemic does not occur then susceptibles continue to build up and the next epidemic will thus be more severe. Note that the incidence peaks of the sporadic epidemics of the stochastic model are higher than those of the biannual epidemics of the continuous model by about a factor of three. The Fourier power spectrum density of the infected time series clearly shows a sequence of peaks corresponding to the annual inflow of susceptibles; Figure 3D. A peak around 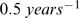 is still visible; however, the peak is now very flat, covering a broad frequency range. The Fourier transform does not appear to provide a very insightful characterization of the epidemic dynamics owing to tall and narrow prevalence peaks that do not occur at very regular intervals.

A more useful approach to revealing periodic patterns in the stochastic time series is a wavelet spectral decomposition. Here we use the Difference-of-Gaussians (DoG) wavelet since it fits well the tall and narrow prevalence peaks of the time series (see Text S1). Figure 3E and 3F show the global spectral decomposition of stochastic orbits in DoG wavelets versus the direct transmissibility 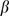 and the re-scaled environmental infectiousness 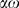, respectively. Each spectrum is an average over 100 wavelet transforms of individual stochastic realizations of the orbit. The white solid lines in Figure 3E and 3F trace the positions of the local peaks in the spectra versus the corresponding system parameters. Note that stochastic time series show periodicity larger than one year (i.e., at ∼2 years and above) for a significantly broader range of 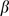 than deterministic time series. Also, note that the dominant periodicity of the stochastic time series changes very little with 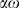, similar to the findings presented in Figure 2F.

### The disease-free/endemic transition

**Figure 4:**
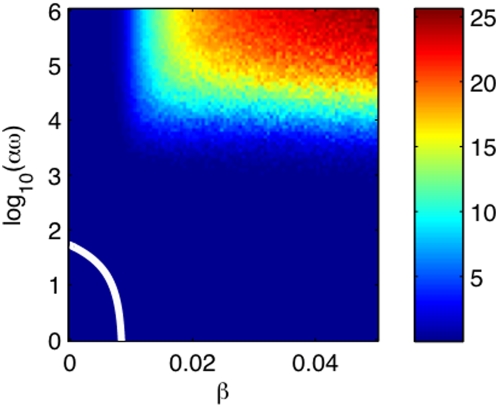
Color map of the time-average of the number of infected 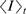 versus the direct transmissibility 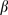 and the environmental infectiousness 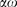. Each colored point is calculated by averaging the results of 100 stochastic realizations. For each realization, a transient of 100 years was discarded and the time average was performed over 200 years. The white line indicates the epidemic threshold of the mean-field model: for parameters in the circled area around the origin there are no epidemics, otherwise epidemics occur. In the Text S1, we present the results of extensive sensitivity analyses.

It is important to distinguish the parameter sets for which AIV is endemic. While many model parameters have empirically-established ranges (e.g., host breeding traits and the duration of infectiousness [21]), the values of other key parameters, such as the direct transmission rate 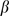 and the environmental infectiousness 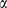 are less certain. Therefore, we explore the plane (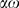, 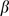) with all the other parameters of the model given in Table 1. For the continuous model, the disease-free state is a periodic attractor with period of one year. This disease-free state loses stability through a transcritical bifurcation which marks the disease-free/endemic transition. Since the bifurcation is codimension one, the transition occurs on a line segment in the (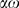, 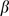) plane; see Figure 4. The segment was obtained by numerically solving for the value of 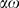 where the transcritical bifurcation of the continuous model with a given value of 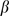 occurs.

For the stochastic model, the disease-free/endemic transition is defined in a more subtle way. The disease-free region is defined by all the parameter sets for which, in all of the realizations of the model, the number of infected 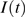 reaches zero in finite time and stays at zero for all subsequent time, irrespective of the initial conditions. The epidemic region is defined by all the parameter sets for which there exist realizations of the model such that, for any moment of time 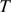, 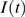 is not zero for all time once 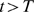. In the disease-free region the probability of an epidemic is zero; however, in the endemic region, the probability of an epidemic increases from zero (close to the boundary with the non-epidemic region) toward one. Therefore, in the case of the stochastic model, it is more difficult to numerically obtain a precise border between the disease-free and the endemic regions. Here we computed the time-average of the infected over 200 years in 100 realizations of the model for a region in the (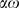, 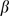) plane; see Figure 4. (A transient of 100 years was discarded for each stochastic realization. Numerical analysis reveals that the results are robust and accurate at these parameters.) Thus, dark blue region corresponds to an epidemic probability of less than ∼1% and encloses the disease-free region. Note that for the probability of a sustained epidemic to be larger than 1%, the re-scaled environmental infectiousness 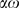 must exceed 10^3^. Simulations did not show sustained epidemics for low or absent environmental transmission.

Because of the way in which the disease-free/endemic transition is defined for the stochastic model, it is difficult to compare the epidemic threshold of the stochastic model with that of the deterministic model. In our case, however, we may expect that they disagree. The mean-field approximation of a stochastic model is obtained in two steps. First, one derives an infinite set of ordinary differential equations that describes how the moments of all orders of the stochastic variables change with time. Second, under the assumption that all stochastic variables are uncorrelated and normally distributed, the set of equations is truncated at the first moment (i.e., *moment closure*) which is the expectation [42]. Disagreement between a stochastic model and its mean-field approximation is expected if the assumptions on normality or correlations are violated. This typically happens when any of the population compartments is small. Here, the disagreement at low numbers of infecteds might be particularly enhanced because of the fact that the continuous model allows for the number of infected birds to be less than one so that we always have two different transmission routes of avian influenza. When the epidemic is at its nadir in the continuous model, the direct transmission rate does not vanish (the number of infected always stays larger than zero even though it may be substantially smaller than one) and thus the the chain of transmission is maintained by both direct and environmental transmission mechanisms. In contrast, in the stochastic model the numbers of infecteds often reaches zero. Therefore, AIV maintenance is exclusively due to environmental transmission. We thus expect that the disease-free region of the stochastic model is larger than that of the deterministic model.

### The interplay between direct and environmental transmission

**Figure 5:**
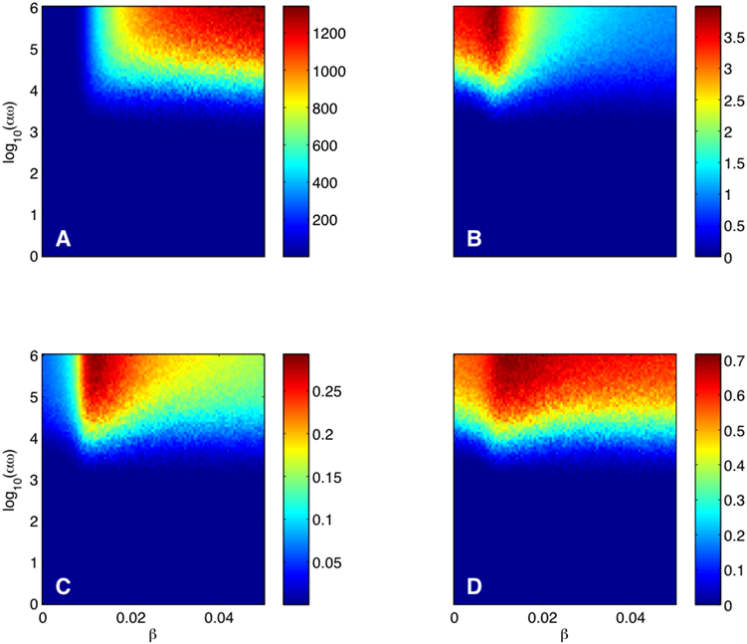
Direct versus environmental transmission. Color maps versus the direct transmissibility 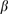 and the environmental infectiousness 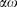 of the time average of the A direct transmission rate; B environmental transmission rate and the average (over stochastic realizations) fraction of time when the C direct transmission is not zero; D environmental transmission is not zero. The simulation details are the same as for Figure 4.

**Figure 6:**
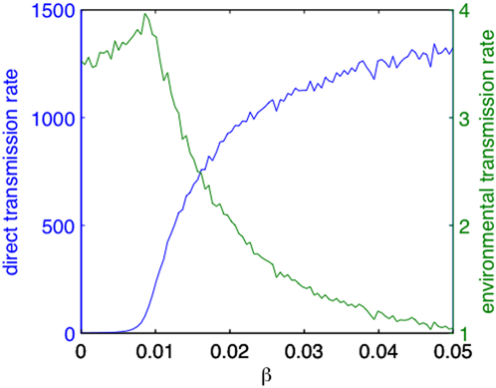
Re-plot of data from Figure 5. Direct and environmental transmission rates versus the direct transmissibility 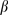 at environmental infectiousness 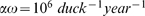.

In Figure 5A and 5B we present the time-averages of the direct and environmental transmission rates, respectively. Note that the environmental transmission rate is two orders of magnitude smaller than the direct transmission rate, yet critical in maintaining the epidemic. The time-average of the direct transmission rate increases with 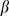 and 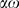, following the pattern of the time-average of the number of infected in Figure 4. However, the time-average of environmental transmission rate has a very different pattern, attaining high values at low values of 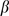 and decreasing at high 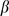; Figure 5B. Another picture of these contrasting patterns is Figure 6. At low 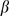, the environmental transmission rate is relatively high and increases with 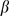 as more infected individuals shed more virus in the environment. A turning point in this scenario happens at 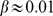 when direct transmission starts to dominate. As the direct and environmental mechanisms of transmission compete for susceptibles, a marked increase in direct transmission results in a decrease of environmental transmission.

A fundamental feature of environmental transmission is the fact that it persists (i.e., does not vanish) even when the number of infecteds (and hence the rate of direct transmission) is zero. As a result, environmental transmission may reignite the epidemic. To contrast the persistence characteristics of direct and environmental transmission, we calculated the average (over stochastic realizations) fraction of time when direct transmission does not vanish (Figure 5C) and environmental transmission does not vanish (Figure 5D). The direct transmission rate vanishes when either 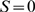 or 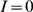 while the environmental transmission rate vanishes when either 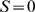 or, quite unlikely, 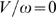. (Here we assumed that the environmental transmission is virtually zero when 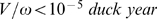. Computations with 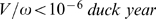 yield very similar results.) From Figure 5C, we obtain that direct transmission at 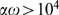 is non-zero at most 30% of the time with a relatively prominent peak at 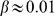. In contrast, the environmental transmission is non-zero at most 70% of the time and the peak is much more shallow over the chosen range of 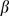. Therefore, even though much smaller than the direct transmission, environmental transmission is much more persistent and may re-ignite the epidemic when there are no infected left.

**Figure 7:**
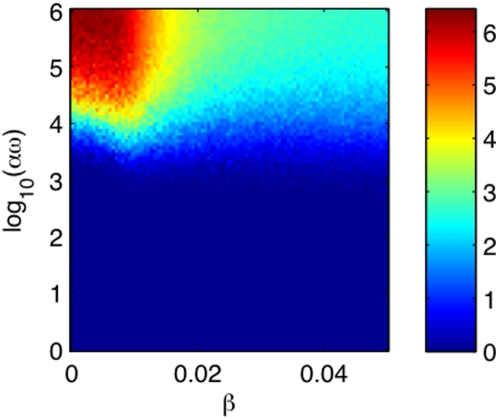
Color map of the time-average of the environmental transmission rate when the epidemic is re-ignited versus the direct transmissibility 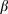 and the environmental infectiousness 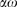. The simulation details are the same as for Figure 4.

An investigation of the time-averaged environmental transmission rate when the epidemic is reignited was performed as follows (Figure 7). Given a stochastic realization of the model, we selected the events where the number of infected increases from zero to one. Say that these events occurred at times 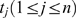 and that the corresponding preceding events occurred at times 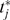 (i.e., for every 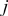, the event at time 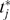 is immediately followed by the event at time 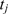). For each event 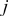, we integrated the environmental transmission rate 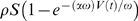 over the time interval (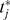,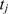). Then, the time-averaged transmission rate when the epidemic is reignited is given by
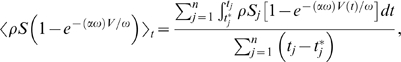
(16)where 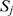 is the number of susceptibles in the time interval (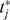,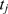) and is a constant. In the analysis presented in Figure 7 we further averaged over 100 realizations of the stochastic model. The pattern in Figure 7 is comparable to that in Figure 5B. Note that the environmental transmission rate that re-ignites the epidemic is less than a factor of two larger than the average.

## Discussion

In this paper, we have explored the epidemiological dynamics and persistence of avian influenza viruses, with a view to understanding the respective roles of environmental transmission and demographic stochasticity. We have found that an 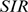 framework that includes seasonal migration, pulsed reproduction and fecal/oral, but not environmental transmission is unable to reproduce the documented recurrent pattern of avian influenza epidemics. The continuous version of the model predicts unrealistic infected populations, with values as low as 10^−8^ individuals (see Text S1), while the stochastic analogue predicts rapid extinction (similar to the depletions of infected in Figure 3B). The unrealistically low infection prevalence is also observed in the continuous model with added environmental transmission Figure 2E and 2F. Including the interaction between the deterministic clockwork of the continuous system and demographic noise is fundamental in obtaining realistic dynamics (with periodicity of 2–4 years), as it is for other infectious diseases; e.g., see [47],[48] and references therein.

In our full hybrid model, we observe that even small levels of environmental transmission (a few cases per year) facilitate AIV persistence. Environmental transmission rates are –on average– hundreds of times smaller than direct transmission rates, yet they appear critical in sustaining the virus. The ability of the pathogen to survive in the environment for a long time before infecting susceptible hosts may thus have profound epidemiological consequences.

The relative influence of environmental transmission for epidemic persistence depends on the population size. If the population is substantially larger than the critical community size, then the number of infecteds does not go to zero in between recurrent epidemics [4],[43],[49] and direct transmission dominates the course of the epidemic. If, however, the population is small and the number of infecteds goes to zero, then environmental transmission is a key factor in sustaining the epidemic. Thus, environmental transmission provides an epidemic persistence mechanism within populations smaller than the critical community size.

Our results hold for low pathogenicity AIV. The extension to high pathogenicity AIVs, as evidenced by outbreaks in tufted ducks and pochards [50], awaits additional empirical information. Another limitation of our model is that we have restricted our consideration to a single immunological subtype that confers life-long immunity. We note that partial cross-immunity in a multi-serotype model would enhance the effective number of susceptibles and, therefore, should be expected to promote persistence. In reference [51], we address the conditions under which environmentally and directly transmitted pathogens may coexist.

The actual mechanism of persistence of avian influenza in wild waterfowl may be complex, including a number of other factors such as spatial and age structures, waning immunity and strain polymorphism leading to immune escape. Several studies address the role of spatial heterogeneity in a general framework. For example, Lloyd and May [52] show in a metapopulation model that persistence of epidemics (asynchrony of within-subpopulation dynamics) occurs only if the immigration in between the subpopulations is small. A more recent and thorough analysis by Hagernaas et al. [53] discussing both oscillatory and non-oscillatory population dynamics arrives at the same conclusion. Further modeling work is needed in order to evaluate the relative contribution of other possible persistence mechanisms.

Further work is also needed to explore our modeling assumption that host populations form (nearly) closed systems. Empirical evidence suggests that the interaction between the Eurasian and American clades of migratory birds is so small (despite overlap in their Alaskan migratory routes) that their exchange of full genome influenza viruses has yet to be documented [54]. While this observation supports our modeling assumption, the data on the smaller scale interaction between flocks of migratory birds within the American continent is insufficient for validation. Alternate modeling assumptions could be explored theoretically.

Using mathematical modeling, we have investigated the role of environmental transmission for the pattern and persistence of avian influenza in wild waterfowl and demonstrated that indeed environmental transmission is a fundamental ingredient for the modeling of this epidemic. The persistence mechanism induced by enviromental transmission raises novel problems of epidemic control since traditional strategies may prove ineffective in the presence of an environmental viral reservoir [55]. Thus, environmental transmission remains a topic of increasing interest in theoretical epidemiology.

## Supporting Information

Text S1 Additional explanations of the parameters, wavelet analysis and further simulations for uncertainty analyses.
(2.05 MB PDF)

## References

[1] Bolker B, Grenfell B (1995). Space, persistence and dynamics of measles epidemics.. Philos Trans R Soc Lond B Biol Sci.

[2] Conlan AJK, Grenfell BT (2007). Seasonality and the persistence and invasion of measles.. Proc Biol Sci.

[3] Keeling MJ, Grenfell BT (1997). Disease extinction and community size: modeling the persistence
of measles.. Science.

[4] Bartlett MS (1957). Measles periodicity and community size (with discussion).. J R Stat Soc Ser A.

[5] Kermack WO, McKendrick AG (1927). A contribution to the mathematical theory of epidemics.. Proc R Soc London.

[6] King AA, Ionides EL, Pascual M, Bouma MJ (2008). Inapparent infections and cholera dynamics.. Nature.

[7] Teyssou R, Rouzic EML (2007). Meningitis epidemics in Africa: a brief overview.. Vaccine.

[8] Greenwood B (2006). Pneumococcal meningitis epidemics in Africa.. Clin Infect Dis.

[9] Rohani P, Earn D, Grenfell B (2000). Impact of immunisation on pertussis transmission in England
& Wales.. Lancet.

[10] Prentice MB, Rahalison L (2007). Plague.. Lancet.

[11] Webster RG, Bean WJ, Gorman OT, Chambers TM, Kawaoka Y (1992). Evolution and ecology of influenza A viruses.. Microbiol Rev.

[12] Widjaja L, Krauss SL, Webby RJ, Xie T, Webster RG (2004). Matrix gene of influenza A viruses isolated from wild aquatic
birds: ecology and emergence of influenza A viruses.. J Virol.

[13] World Health Organization Cumulative number of confirmed human cases of avian influenza
A/(H5N1) reported to WHO.

[14] Krauss S, Walker D, Pryor SP, Niles L, Chenghong L (2004). Influenza A viruses of migrating wild aquatic birds in North
America.. Vector Borne Zoonotic Dis.

[15] Sharp GB, Kawaoka Y, Wright SM, Turner B, Hinshaw V (1993). Wild ducks are the reservoir for only a limited number of
influenza A subtypes.. Epidemiol Infect.

[16] Stallknecht DE, Kearney MT, Shane SM, Zwank PJ (1990). Effects of pH, temperature, and salinity on persistence of avian
influenza viruses in water.. Avian Dis.

[17] Brown JD, Stallknecht DE, Beck JR, Suarez DL, Swayne DE (2006). Susceptibility of North American ducks and gulls to H5N1 highly
pathogenic avian influenza viruses.. Emerg Infect Dis.

[18] Stallknecht DE, Brown JD (2007). Wild birds and the epidemiology of avian influenza.. J Wildl Dis.

[19] Hinshaw VS, Webster RG, Turner B (1979). Water-bourne transmission of influenza A viruses?. Intervirology.

[20] Stallknecht DE, Shane SM, Kearney MT, Zwank PJ (1990). Persistence of avian influenza viruses in water.. Avian Dis.

[21] Brown JD, Goekjian G, Poulson R, Valeika S, Stallknecht DE (2008). Avian influenza virus in water: infectivity is dependent on pH,
salinity, and temperature.. Vet Microbiol.

[22] D'Souza DH, Sair A, Williams K, Papafragkou E, Jean J (2006). Persistence of caliciviruses on environmental surfaces and their
transfer to food.. Int J Food Microbiol.

[23] Henning J, Meers J, Davies PR, Morris RS (2005). Survival of rabbit haemorrhagic disease virus (RHDV) in the
environment.. Epidemiol Infect.

[24] Pascual M, Rodo X, Ellner SP, Colwell R, Bouma MJ (2000). Cholera dynamics and el Niño-southern oscillation.. Science.

[25] Blanchong JA, Samuel MD, Goldberg DR, Shadduck DJ, Lehr MA (2006). Persistence of Pasteurella multocida in wetlands following avian
cholera outbreaks.. J Wildl Dis.

[26] Roper MH, Vandelaer JH, Gasse FL (2007). Maternal and neonatal tetanus.. Lancet.

[27] Xiao Y, Bowers RG, Clancy D, French NP (2007). Dynamics of infection with multiple transmission mechanisms in
unmanaged/managed animal populations.. Theor Popul Biol.

[28] Webb CT, Brooks CP, Gage KL, Antolin MF (2006). Classic flea-borne transmission does not drive plague epizootics
in prairie dogs.. Proc Natl Acad Sci U S A.

[29] Miller MW, Hobbs NT, Tavener SJ (2006). Dynamics of prion disease transmission in mule deer.. Ecol Appl.

[30] Anderson RM, Donnelly CA, Ferguson NM, Woolhouse ME, Watt CJ (1996). Transmission dynamics and epidemiology of BSE in British cattle.. Nature.

[31] Field H, Young P, Yob JM, Mills J, Hall L (2001). The natural history of Hendra and Nipah viruses.. Microbes Infect.

[32] Joh RI, Wang H, Weiss H, Weitz JS (2008). Dynamics of indirectly transmitted infectious dynamics of
indirectly transmitted infectious diseases with immunological threshold.. Bull Math Biol.

[33] Capasso V, Paveri-Fontana SL (1979). A mathematical model for the 1973 cholera epidemic in the
European Mediterranean region.. Rev Epidemiol Sante Publique.

[34] Codeco CT (2001). Endemic and epidemic dynamics of cholera: the role of the aquatic
reservoir.. BMC Infect Dis.

[35] Pascual M, Bouma MJ, Dobson AP (2002). Cholera and climate: revisiting the quantitative evidence.. Microbes Infect.

[36] Hartley DM, Morris JGJ, Smith DL (2006). Hyperinfectivity: a critical element in the ability of V.
cholerae to cause epidemics?. PLoS Med.

[37] Jensen MA, Faruque SM, Mekalanos JJ, Levin BR (2006). Modeling the role of bacteriophage in the control of cholera
outbreaks.. Proc Natl Acad Sci U S A.

[38] Codeco CT, Lele S, Pascual M, Bouma M, Ko AI (2008). A stochastic model for ecological systems with strong nonlinear
response to environmental drivers: application to two water-borne diseases.. J R Soc Interface.

[39] Aczel J, Dhombres J (2008). Functional Equations in Several Variables.

[40] Mollison D (1991). Dependence of epidemic and populations velocities on basic
parameters.. Math Biosci.

[41] Gillespie DT (1976). A general method for numerically simulating the stochastic time
evolution of coupled chemical reactions.. J Comput Phys.

[42] Bailey NT (1975). The Mathematical Theory of Infectious Diseases..

[43] Keeling M, Rohani P (2007). Modeling Infectious Diseases in Humans and Animals.

[44] Otto SP, Day T (2007). A Biologist's Guide to Mathematical Modeling in Ecology and
Evolution.

[45] Aldroubi A, Unser M (1996). Wavelets in Medicine and Biology.

[46] Strogatz SH (1994). Nonlinear Dynamics and Chaos: With Applications to Physics, Biology,
Chemistry and Engineering.

[47] Rohani P, Keeling M, Grenfell B (2002). The interplay between determinism and stochasticity in childhood
diseases.. Am Nat.

[48] Alonso D, McKane AJ, Pascual M (2007). Stochastic amplification in epidemics.. J R Soc Interface.

[49] Nasell I (2005). A new look at the critical community size for childhood
infections.. Theor Popul Biol.

[50] Keawcharoen J, van Riel D, van Amerongen G, Bestebroer T, Beyer WE (2008). Wild ducks as long-distance vectors of highly pathogenic avian
influenza virus (H5N1).. Emerg Infect Dis.

[51] Breban R, Drake JM, Rohani P (2009). A general multi-strain model with environmental transmission:
invasion conditions for the disease-free and endemic states.. Preprint.

[52] Lloyd AL, May RM (1996). Spatial heterogeneity in epidemic models.. J Theor Biol.

[53] Hagenaars TJ, Donnelly CA, Ferguson NM (2004). Spatial heterogeneity and the persistence of infectious diseases.. J Theor Biol.

[54] Krauss S, Obert CA, Franks J, Walker D, Jones K (2007). Influenza in migratory birds and evidence of limited
intercontinental virus exchange.. PLoS Pathog.

[55] Rohani P, Breban R, Stallknecht DE, Drake JM (2008). Environmental transmission of avian influenza viruses and its
implications for disease control.. Preprint.

[56] Sargeant AB, Raveling DG, Batt BDJ, Afton AD, Anderson MG, Ankney CD, Johnson DH (1992). Mortality during the breeding season.. Ecology and Management of Breeding Waterfowl.

[57] Kear J (2005). Ducks, Geese, and Swans, volume 16 of Bird Families of the World.

[58] Webster RG, Yakhno M, Hinshaw VS, Bean WJ, Murti KG (1978). Intestinal influenza: replication and characterization of
influenza viruses in ducks.. Virology.

