# Supplementary material for: The Role of Environmental Transmission in Recurrent Avian Influenza Epidemics
Source: PLoS Comput Biol. 2009 Apr 10;5(4):e1000346. doi: 10.1371/journal.pcbi.1000346 (PMC2660440; doi:10.1371/journal.pcbi.1000346)
Supplement: Text S1 — Additional explanations of the parameters, wavelet analysis and further simulations for uncertainty analyses. (2.05 MB PDF) [file pcbi.1000346.s001.pdf]

The role of environmental transmission in recurrent avian influenza epidemics

Supporting Information

Romulus Breban[1,*], John M. Drake[1], David E. Stallknecht[2] & Pejman Rohani[1,3]

[1] *Odum School of Ecology,*
[2] *Southeastern Cooperative for Wildlife Disease Study,*
[3] *Center for Tropical and Emerging Global Diseases,*
University of Georgia, Athens, GA 30602, USA.

February 10, 2009

Contents

1 **Parameters of the model** 2

2 **The deterministic model without environmental transmission** 3

3 **Difference-of-Gaussians wavelet analysis** 4

4 **Numerical explorations of pattern robustness** 5

*Present Address: Unité d'Epidémiologie des Maladies Emergentes, Institut Pasteur, Paris, France.

1 Parameters of the model

Here we discuss further details of the parameterization of the model. We provide a description of the parameters that we use in the main text and their corresponding values in Table 1 below. Parameters that deserve special consideration are the habitat carrying capacity N_d, the direct transmissibility β, the exposure rate ρ, and the re-scaled environmental infectiousness $\alpha\omega^\dagger$. These are as follows:

- The value of N_d is chosen such that the model in absence of infection yields ∼5,000 to 6,000 susceptibles, in agreement with observed duck population sizes in the wild [1].
- β is a study parameter. Defining $R_0^{\text{direct}} = S\beta/(\mu+\gamma)$, a range of β between 0 and 0.05 corresponds to a fairly large range of R_0^{direct} between 0 and 5.74. The value of 0.15 that we use for β in the simulations described in Figs. 2, 3 of the main text corresponds to an $R_0^{\text{direct}} \sim 1.6$.
- The exposure rate ρ is given by the drinking rate of the duck (ranging from 2.5×10^3 to 2.5×10^4 $liters/year$ [2]) divided by the volume of water that dilutes the virus. Since we are not modeling a particular pool of water, the choice of ρ remains somehow arbitrary. Therefore, in Sec. 4 of this supplement, we explore the robustness of our results with varying ρ.
- $\alpha\omega$ is a study parameter that we vary over six orders of magnitude, as little is known about this parameter.

In the following sections of this supplement, we present further numerical explorations that support our statements in the main text.

Table 1: The parameters of the model.

Symbol	Definition	Value/Range	Unit
N_d	habitat capacity	3000	$duck$
λ	duck fecundity	2	
β	direct transmissibility	0-0.05	$duck^{-1}year^{-1}$
ρ	exposure rate	10^{-3}	$year^{-1}$
α	environmental infectiousness		$virion^{-1}$
ω	virus shedding rate	10^5-10^6	$virion/duck/day$
$\alpha\omega$	re-scaled environmental infectiousness	1-10^6	$duck^{-1}year^{-1}$
μ	natural death rate	1/3	$year^{-1}$
γ	recovery rate	52	$year^{-1}$
η_B^b	virus clearance rate in the breeding grounds during the summer	5	$year^{-1}$
η_W^b	virus clearance rate in the breeding grounds during the winter	1.3	$year^{-1}$
η_W^w	virus clearance rate in the wintering grounds during the winter	5	$year^{-1}$
η_B^w	virus clearance rate in the wintering grounds during the summer	50	$year^{-1}$

† α may be calculated using the values that we provide for $\alpha\omega$ and ω.

2 The deterministic model without environmental transmission

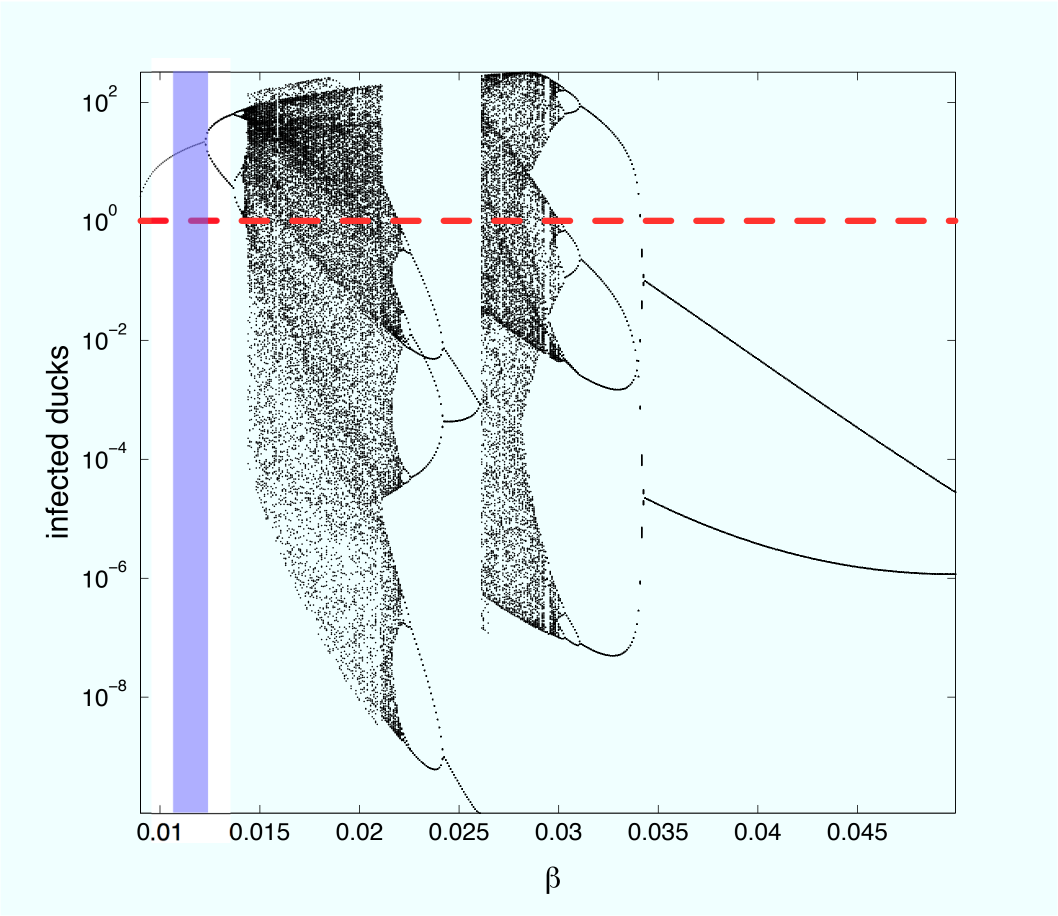

Figure 1: Bifurcation diagram of a deterministic SIR model that includes migration, seasonality, pulse reproduction, but no environmental transmission (i.e., $\rho = 0$, all other parameter values are as in Table 1) versus the direct transmissibility β. The orbits are sampled yearly, at the end of the wintering season. Note that for a large range of β, the model shows numbers of infected ducks less than one (i.e., the area below the dotted red line) which elude a biological interpretation. All orbits sampled for Fig. 1 have at least one point below 22. If, we discard all orbits with points below (let us say) 10 as being plagued by the "atto-fox" phenomenon (as the fractional parts of these predictions may represent a significant percent from their corresponding integer parts), then we are left with a very narrow β-interval for which our continuous model has biological interpretation (i.e., the blue region). It is unlikely that this model with periodicity of 1-2 years would be validated by AIV epidemiological data from the wild habitat. In fact, prevalence data exhibits outbreaks with a 2-4 year periodicity and possibility of extinction between the outbreaks. Since we are particularly interested in the processes of extinction and persistence of AIV, we refined our study by constructing a stochastic model, where the host population variables are integer-valued.

3 Difference-of-Gaussians wavelet analysis

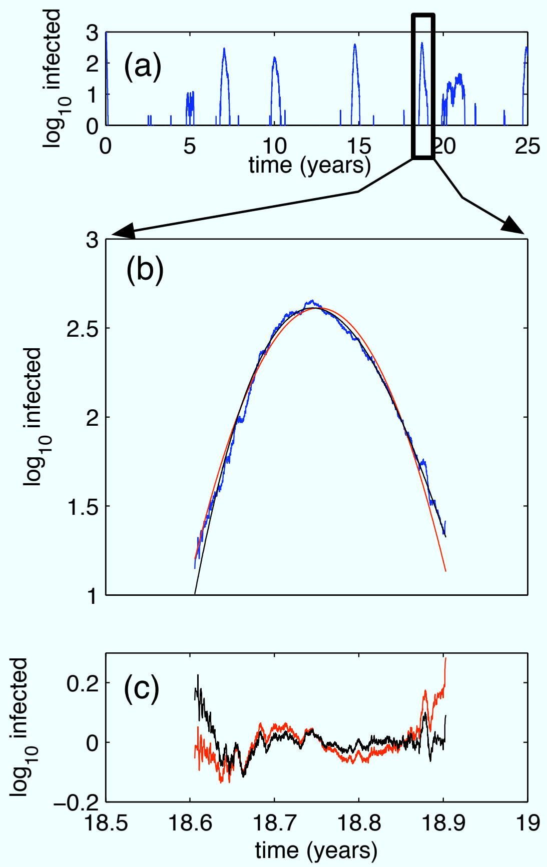

Figure 2: Fit of outbreak prevalence profile with Difference-of-Gaussians (DoG) wavelet. Given the prevalence time series presented in panel (a), we selected a prevalence peak and we fitted the logarithm of the prevalence with both a quadratic and a cubic polynomial; see panel (b) where the simulation is in blue, the quadratic fit ($-0.2274z^2 + 0.0512z + 2.609$, where $z = (t - 18.746)/0.05887$) is in red and the cubic fit ($0.02088z^3 - 0.2351z^2 - 0.004106z + 2.612$) is in black. Panel (c) plots the corresponding residuals. Note that the residuals of the quadratic and the cubic fits are very similar (also note that the cubic coefficient is small), suggesting that the prevalence curve can be parsimoniously described very well by a Gaussian. Since a DoG wavelet where the subtracted Gaussian is very flat approaches a Gaussian, the outbreak prevalence profile is fitted very well by a DoG wavelet.

4 Numerical explorations of pattern robustness

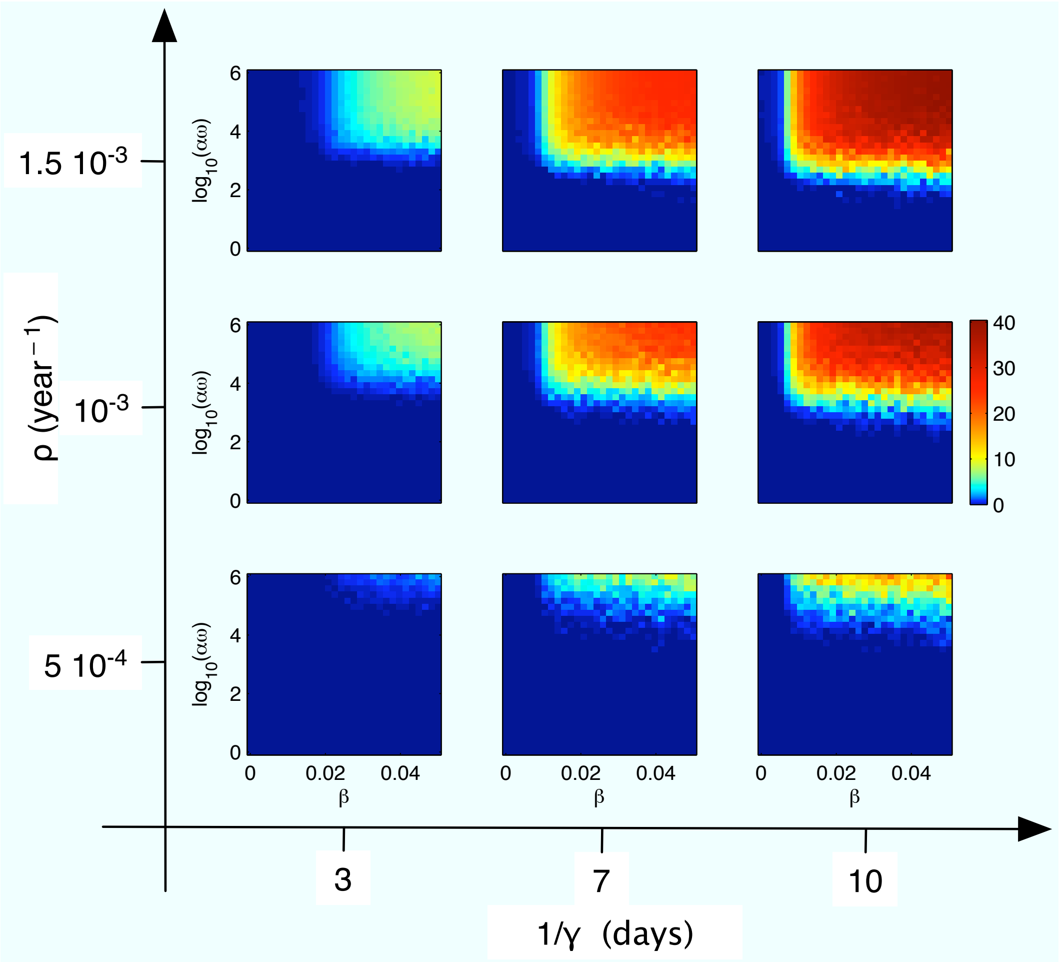

Figure 3: Color maps of the time-average of the number of infected $\langle I \rangle_t$ versus the direct transmissibility β and the environmental infectiousness $\alpha\omega$ at different values of the recovery rate γ and the exposure rate ρ. All the other parameters are listed in the Table 1. Each colored point is calculated by averaging the results of 100 stochastic realizations. For each realization, a transient of 100 years was discarded and the time average was performed over 200 years. The pattern in the central panel is further discussed in the main text. The time-average of the number of infected does change with the parameters γ and ρ. Note, however, that its pattern in the $(\alpha\omega, \beta)$ plane qualitatively stays the same even though the parameters γ and ρ vary over a large range.

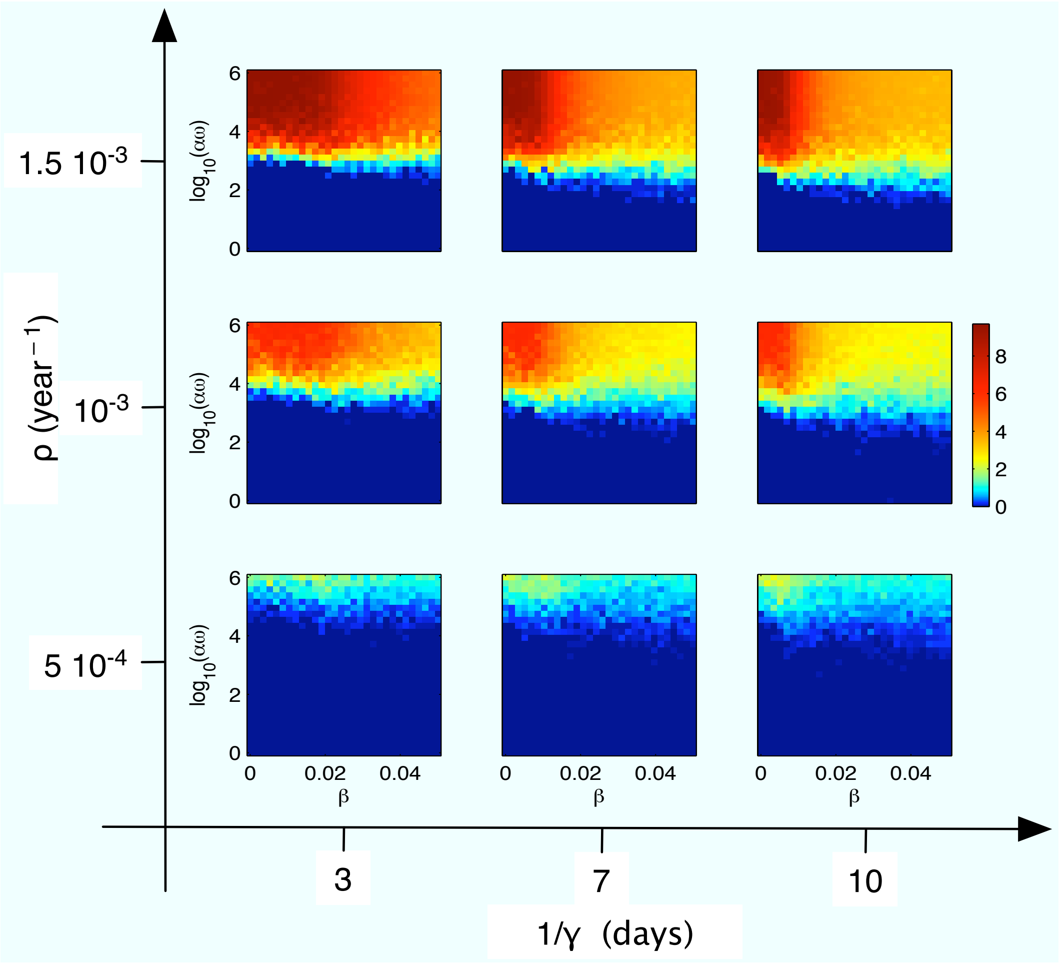

Figure 4: Color maps of the time-average of the environmental transmission rate when the epidemic is re-ignited versus the direct transmissibility β and the environmental infectiousness $\alpha\omega$ at different values of the recovery rate γ and the exposure rate ρ. All the other parameters are listed in the Table 1. The simulation details are the same as for Fig. 3. The pattern in the central panel is further discussed in the main text. Note that the displayed pattern remains robust over a large range of γ and ρ.

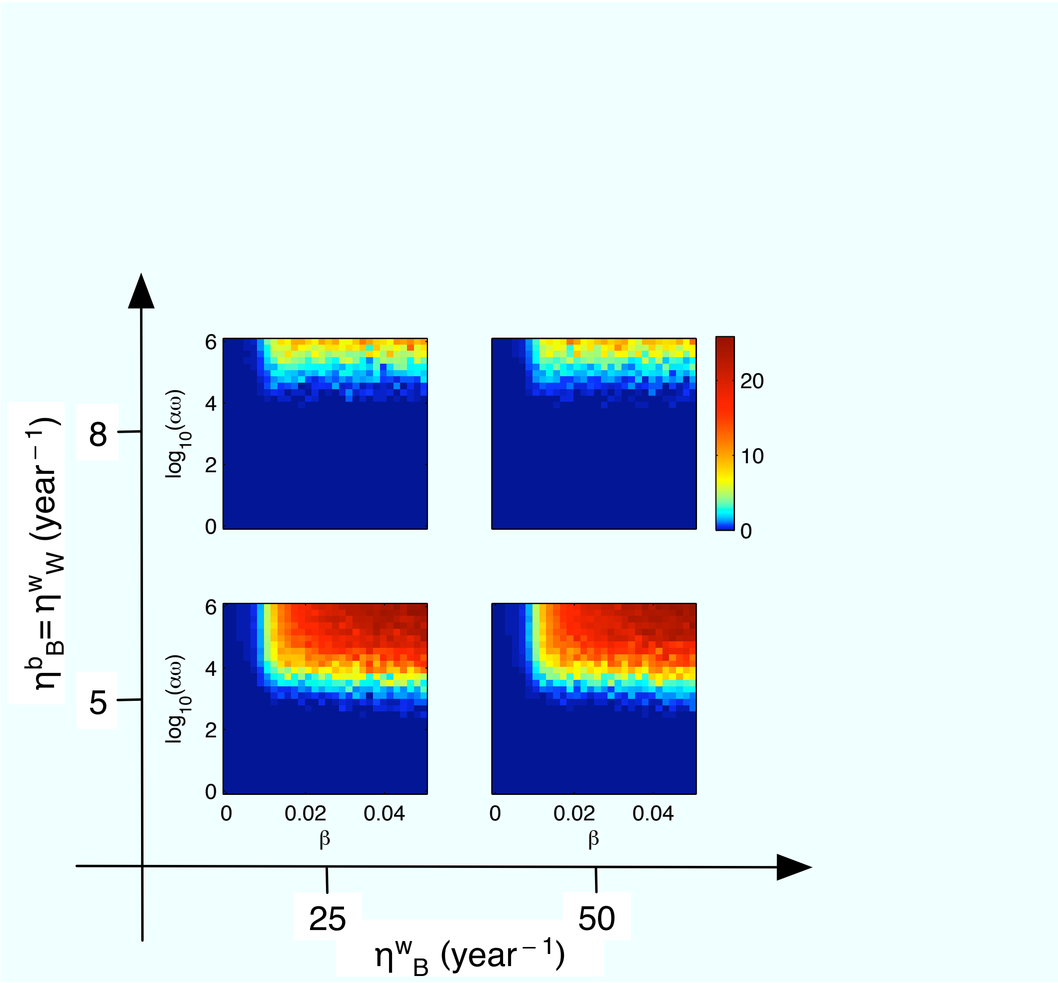

Figure 5: Color maps of the time-average of the number of infected $\langle I \rangle_t$ versus the direct transmissibility β and the environmental infectiousness $\alpha\omega$ at different values of the viral persistence at the breeding site in the summer η_B^b (which we chose to be equal to the viral persistence rate at the wintering site in the winter η_W^w) and the viral persistence rate at the wintering grounds in the summer η_B^w. All the other parameters are listed in the Table 1. The simulation details are the same as for Fig. 3. The pattern in the low right panel is further discussed in the main text. Again, we notice that the displayed pattern is robust with changing parameters.

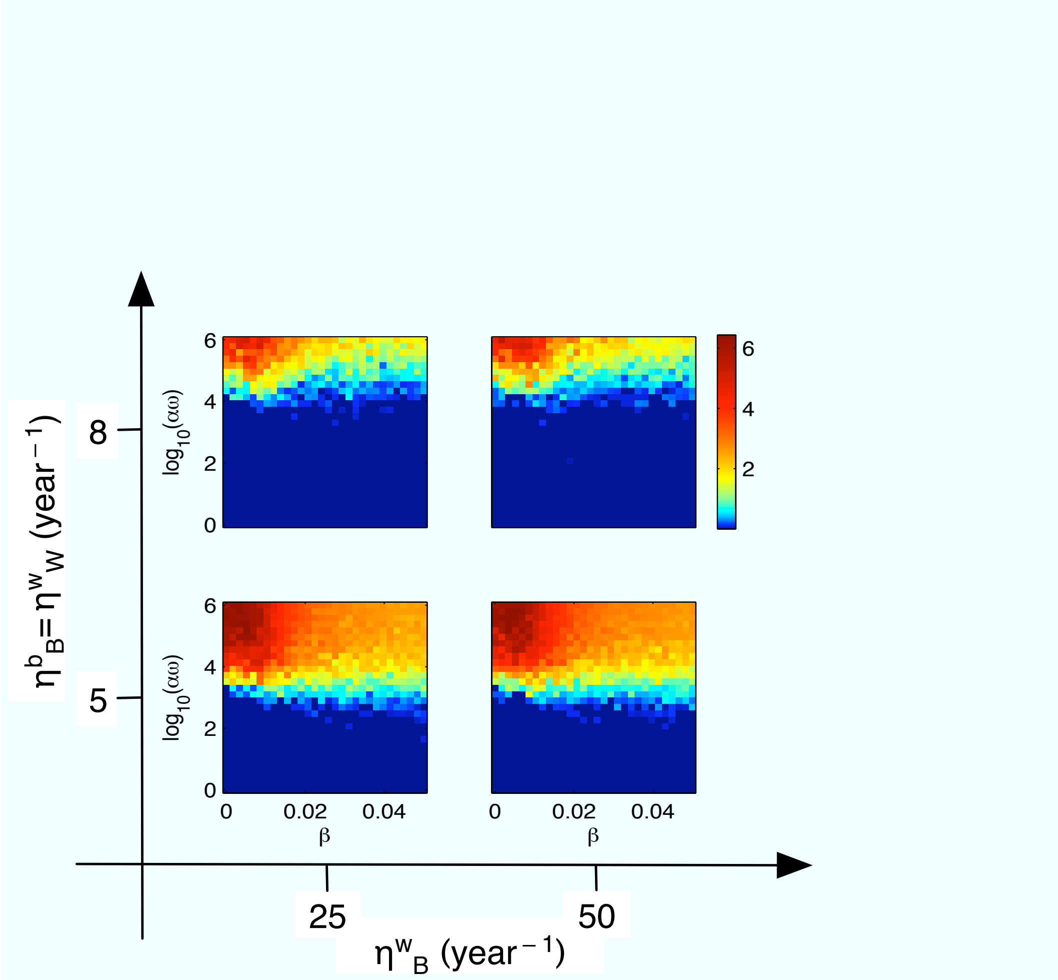

Figure 6: Color maps of the time-average of the environmental transmission rate when the epidemic is re-ignited versus the direct transmissibility β and the environmental infectiousness $\alpha\omega$ at different values of the viral persistence at the breeding site in the summer η_B^b (which we chose to be equal to the viral persistence rate at the wintering site in the winter η_W^w) and the viral persistence rate at the wintering grounds in the summer η_B^w. All the other parameters are listed in the Table 1. The simulation details are the same as for Fig. 3. The pattern in the low right panel is further discussed in the main text. Note that the displayed pattern remains robust with changing parameters.

References

[1] U S Fish and Wildlife Service (2008) Waterfowl population status, 2008. Technical report, Department of the Interior, Washington, DC, USA.

[2] Bennett DC, Hughes MR (2003) Comparison of renal and salt gland function in three species of wild ducks. J Exp Biol 206:3273–3284.
